# Diagnostic technologies for circulating tumour cells and exosomes

**DOI:** 10.1042/BSR20150180

**Published:** 2016-02-03

**Authors:** Huilin Shao, Jaehoon Chung, David Issadore

**Affiliations:** * Institute of Molecular and Cell Biology, Agency for Science, Technology and Research, 61 Biopolis Drive, Singapore 138673, Singapore; † National Neuroscience Institute, 11 Jalan Tan Tock Seng, Singapore 308433, Singapore; ‡ Center for Systems Biology, Massachusetts General Hospital, Harvard Medical School, Boston, MA 02114, U.S.A.; § Institute of Microelectronics, Agency for Science, Technology and Research, 11 Science Park Road, Singapore 117685, Singapore; ║ School of Engineering and Applied Sciences, University of Pennsylvania, Philadelphia, PA 19104, U.S.A.

**Keywords:** cancer, circulating tumour cells, exosomes, molecular diagnostics, technologies

## Abstract

Circulating tumour cells (CTCs) and exosomes are promising circulating biomarkers. They exist in easily accessible blood and carry large diversity of molecular information. As such, they can be easily and repeatedly obtained for minimally invasive cancer diagnosis and monitoring. Because of their intrinsic differences in counts, size and molecular contents, CTCs and exosomes pose unique sets of technical challenges for clinical translation–CTCs are rare whereas exosomes are small. Novel technologies are underway to overcome these specific challenges to fully harness the clinical potential of these circulating biomarkers. Herein, we will overview the characteristics of CTCs and exosomes as valuable circulating biomarkers and their associated technical challenges for clinical adaptation. Specifically, we will describe emerging technologies that have been developed to address these technical obstacles and the unique clinical opportunities enabled by technological innovations.

## INTRODUCTION

**Figure 1:**
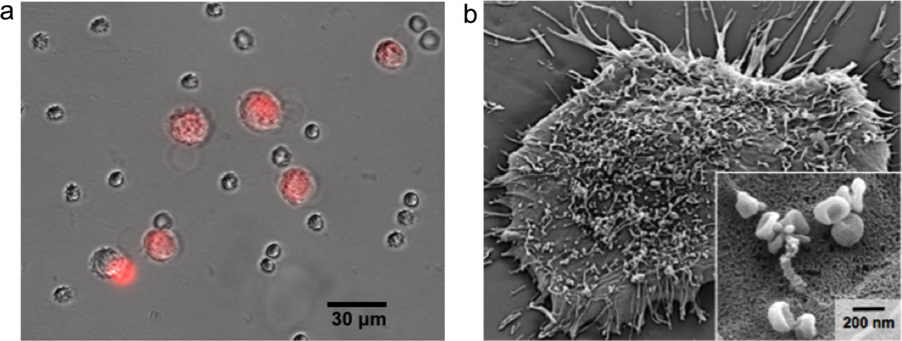
Circulating biomarkers (**a**) Circulating tumour cells (CTCs). Human epidermoid cancer cells (red) in the presence of leucocytes. (Adapted with permission from [17]: Chung, J., Shao, H., Reiner, T., Issadore, D., Weissleder, R. and Lee, H. (2012) Microfluidic cell sorter (muFCS) for on-chip capture and analysis of single cells. Adv. Healthc. Mater. **1**, 432–436. Copyright 2012 John Wiley and Sons Inc.) (**b**) Exosomes. Scanning electron image of a primary human glioblastoma cell confirms the avid release of membrane vesicles by cells. Higher magnification insert shows that the vesicles on the cell surface assume typical saucer-shaped characteristics of exosomes. (Adapted with permission from [21]: Shao, H., Chung, J., Balaj, L., Charest, A., Bigner, D.D., Carter, B.S., Hochberg, F.H., Breakefield, X.O., Weissleder, R. and Lee, H. (2012) Protein typing of circulating microvesicles allows real-time monitoring of glioblastoma therapy. Nat. Med. **18**, 1835–1840. Copyright 2012 Nature Publishing Group.)

Many solid cancers shed materials into the systemic circulation. These include cells (circulating tumour cells; CTCs) [1,2] and extracellular vesicles [3–5], such as exosomes [6,7] and other types of sub-cellular membrane vesicles (Figure 1). Many of these materials exist in easily accessible bodily fluids, such as peripheral whole blood, peritoneal or pleural effusions, and carry large diversity of molecular information, including proteins, nucleic acids and lipids [8,9]. These shed materials thus represent valuable circulating biomarkers for tumour diagnosis, staging and treatment monitoring.

CTCs exfoliated from primary tumours have been identified as a valuable disease indicator–both CTC concentration and number changes can be used as prognostic and predictive markers, although the molecular contents can serve as clinical indices for tumour stratification [1,10]. Exosomes, on the other hand, have recently emerged as an abundant and diverse source of circulating biomarkers. Exosomes are membrane-bound phospholipid vesicles (30–150 nm in diameter) actively secreted by a variety of mammalian cells, especially dividing cancer cells, and carry diverse molecular contents [6,11].

Immense interests have been directed towards these circulating biomarkers for minimally invasive testing. Although tissue biopsies remain the gold standard for molecular evaluation, their invasiveness and limited sampling present challenges in clinical management, especially in monitoring tumour spatial heterogeneity, temporal evolution and treatment response [12]. Circulating biomarkers present significant advantages to circumvent such challenges–circulating biomarkers can be easily and repeatedly obtained to provide a minimally invasive reflection of tumour molecular information. Despite such clinical potential, due to the diverse nature of these circulating biomarkers, their isolation and quantitative analyses remain technically challenging, particularly because each type of circulating biomarkers poses unique technical challenges for clinical translation.

Leveraging on novel isolation and sensing mechanisms, significant engineering developments are underway to overcome these obstacles. Miniaturized and sophisticated systems have been designed to facilitate sensitive analyses of circulating biomarkers from small sample volumes, while requiring minimal sample preparation. Specifically, multiple microfluidic systems have been developed to rapidly enrich for these biomarkers and detect them through nanotechnology-inspired sensing mechanisms [13–29]. These emerging technologies not only address the technical challenges associated with diverse circulating biomarkers but also help to unleash many unique clinical opportunities for rapid and minimally invasive molecular testing.

## CTCS FOR CANCER DETECTION

CTCs are cells shed off from a primary tumour into the circulation (Figure 1a) [30]. Although their existence has been long known since the nineteenth century, it is only with recent technological developments that extensive molecular characterization of CTCs has been achieved. The presence of CTCs in blood is considered an essential step in establishing distant metastases; recent studies have shown that a subset of CTCs have tumour-forming capacity [31,32]. Because disseminated malignancies are responsible for the majority of cancer-related deaths [33], CTCs have received tremendous attention as a circulating biomarker.

### CTCs as disease markers

CTCs have been identified in many different cancers. It is now widely accepted that CTCs found in peripheral blood originate from solid tumours and are involved in the haematogenous metastatic migration of solid tumours to distant sites [30]. The metastatic cascade is a complex process [34]. First, the CTC must detach from the primary tumour and intravasate to enter the vasculature. A leading hypothesis on processes underlying the intravasation involves epithelial-to-mesenchymal transition (EMT), during which cancer cells revert to a state resembling the mobile cells in the developing embryo [35]. Once inside the circulation, the CTC must evade immune detection and extravasate into target tissues. Finally, to form a distant metastasis, the CTC need to colonize and engraft successfully by recruiting and forming new blood vessels, a process known as neoangiogenesis [34]. The successful formation of a metastatic lesion thus depends on the CTC's ability to adapt, survive and induce neoangiogenesis, all of which could be used as potential clinical indicators for detecting and prognosticating cancers [1,2].

Indeed, recent studies have shown that different physical and molecular properties of CTCs can be correlated to cancer type, staging and treatment response. For example, physical attributes of CTCs (such as counts [36], microstructures [37] and clustering [38]) are valuable prognostic indicators. CTC counts and concentration changes have been associated with tumour burden [36]. More recently, studies have shown that multicellular CTC clusters may harbour significantly increased (>20-fold) metastatic potential [38]. Importantly, the presence of CTC clusters was associated with a shorter disease-free progression and reduced overall survival in metastatic breast cancer patients and prostate cancer patients.

CTCs may also be characterized by specific molecular signatures. Recent studies have investigated the use of surface antigens for CTC enrichment and tumour typing: epithelial cell adhesion molecule (EpCAM) is ubiquitously expressed on the surface of epithelial carcinomas and remains the most commonly used marker for enriching and detecting CTCs of breast [39], colon [40] and prostate [41] origins; membrane-bound mucin (MUC1) has been used to detect CTCs of breast [42] and ovary carcinomas [43]; and the ephrin receptor (EphB4) is strongly over-expressed in advanced head and neck cancers [44]. In addition to surface proteins, initial data comparing CTCs with primary and metastatic tumours have indicated a large number of similar genetic mutations between primary tumour and CTCs [45,46], making CTCs valuable surrogate markers of the primary tumour. As prognostics markers, specific CTC signatures have also been identified to associate with a high metastatic capability. For example, a brain metastatic signature of breast cancer CTCs has been recently reported [47].

### CTC challenges and detection technologies

Although CTCs are been used in many clinical trials, their detection and characterization remain technically challenging. CTCs are extremely rare (as few as one cell per 10^9^ haemotologic cells [48]); their isolation and characterization from blood thus require stringent and effective enrichment against a complex biological background. As previously mentioned, the extravasation of CTCs in the metastatic cascade requires the cells to adapt. Carcinoma cells undergoing EMT have reduced expression of epithelial surface markers, which are commonly used for positive enrichment of CTCs, and can result in potential false-negative findings [49]. Moreover, CTC viability in the circulation is rather limited and, in patients with breast cancer, their half-life was estimated to be in the range of 1–2 h [50]. Hence, CTCs need to be enriched, processed and measured rapidly from large volumes of fresh blood samples.

**Figure 2:**
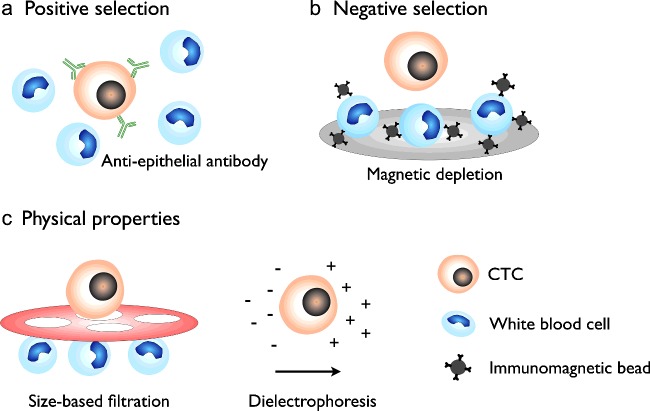
CTC enrichment technologies (**a**) Positive selection and detection of CTCs by specific epithelial markers which are expressed on CTCs but absent on mesenchymal white blood cells. Such enrichment typically requires assumptions on the nature of CTCs and introduces selection bias. (**b**) In negative selection, abundant blood cells are targeted and depleted, commonly through immunomagnetic removal, leading to an overall CTC enrichment. Negative depletion overcomes the selection bias associated with positive selection. (**c**) CTCs can also be positively and/or negatively enriched on the basis of their physical properties (e.g., size and charge). Size-based filtration isolates larger CTCs (or CTC clusters) from smaller blood cells. Dielectrophoresis separates CTCs from blood cells based on their differences in electric charges when dispersed in a particular medium conductivity.

New technology platforms have permitted the reliable capture and characterization of CTCs. To improve isolation yield and purity, two general mechanisms of CTC enrichment have been developed and implemented in various miniaturized devices: positive and negative selection (Figure 2). In positive selection, CTCs are directly isolated from blood. This could be done through protein-based technologies that rely on immunocapturing of CTC epithelial antigens (Figure 2a). Epithelial markers (e.g., EpCAM) are expressed on carcinomas but absent on mesenchymal leucocytes, and are frequently used to enrich and/or distinguish cancer cells from normal blood cells [13–16,18]. Such positive selection of CTCs, however, requires assumptions on the nature of CTCs (e.g., molecular markers) in individual blood samples and introduces selection bias. This bias is avoided by negative selection in which abundant leucocytes are depleted through immunomagnetic targeting and removal against CD45 and other leucocyte antigens (Figure 2b) [51,52]. In addition to molecular selection, physical properties of CTCs (e.g., size [17,19,20] and electric charges [53,54]) can also be used to positively and/or negatively enrich cancer cells from blood cells (Figure 2c).

#### Microfluidic enrichment

In 2012, a rapid microfluidic cell sorter (μFCS) device was developed for the isolation and enrichment of single CTCs [17]. The μFCS employs a modified weir-style physical barrier to separate and capture CTCs from unprocessed whole blood based on their size difference. The operation is performed in a continuous-flow manner, processing large volumes of samples at high flow rates (up to 20 ml/h), without clogging or pressure buildup. The captured cells can then be analysed *in situ* for comprehensive molecular profiling, as well as cultured on-chip for subsequent drug screening and genetic analyses.

More recently, a dedicated microfluidic chip was designed to capture clusters of CTCs, rather than single cells. This platform, called Cluster-Chip, exploits the physical properties of cell clusters, independently of tumour-specific markers from unprocessed blood [20]. The chip consists of rows of triangular microposts arranged in a way that cell clusters are efficiently captured through bifurcating traps, while single CTCs and blood cells will flow through. Using the Cluster-Chip, the authors identified CTC clusters in >30% of patients with metastatic cancers. Interestingly, RNA sequencing of CTC clusters also identified tissue-derived macrophages within the clusters.

#### Magnetic detection

Magnetic sensing offers many advantages for CTC detection as human samples are naturally devoid of ferromagnetic background. By tagging molecular markers with specific magnetic nanoparticles (MNPs), such detection mechanism enables minimal sample processing, potentially enabling point-of-care molecular testing.

**Figure 3:**
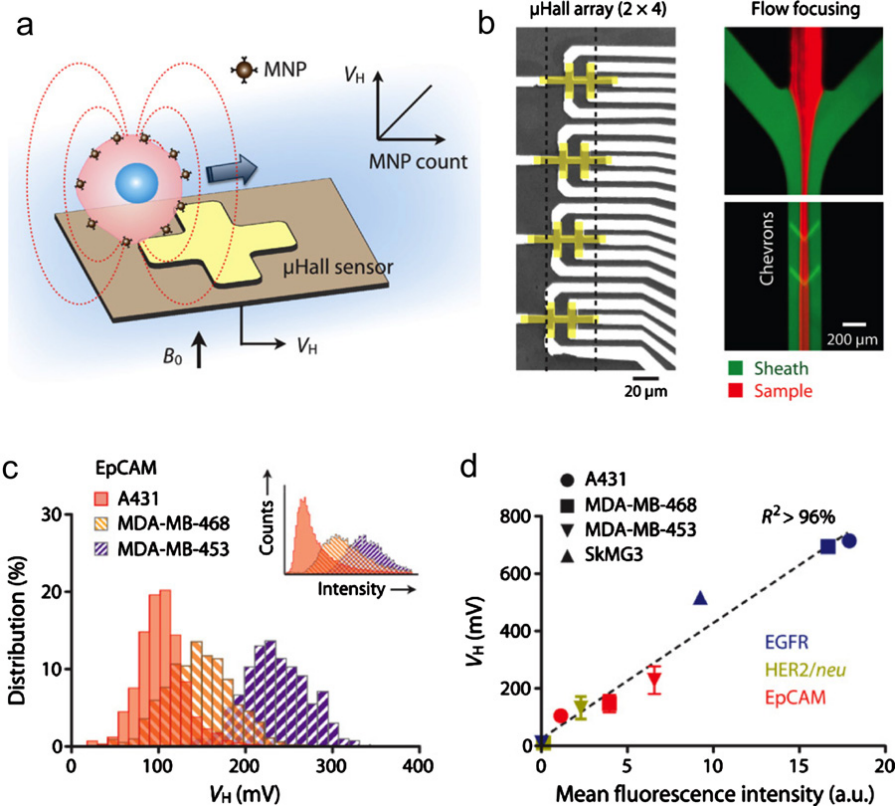
μHall sensor for single cell detection in flow condition (**a**) Each cell, targeted with MNPs, generates magnetic fields that are detected by the μHall sensor. The Hall voltage (*V_H_*) is proportional to the MNP counts. *B*_0_, external magnetic field. (**b**) Eight μHall sensors are arranged into an overlapping 2×4 array across the fluidic channel width. The dotted lines indicate the confined sample flow, which was achieved by flow focusing. (**c** and **d**) The μHall system measured the expression levels of EpCAM (c) and other biomarkers (**d**) in different cell lines, which agreed with measurements by flow cytometry. (Reproduced with permission from [18]: Issadore, D., Chung, J., Shao, H., Liong, M., Ghazani, A.A., Castro, C.M., Weissleder, R. and Lee, H. (2012) Ultrasensitive clinical enumeration of rare cells *ex vivo* using a micro-hall Detector. Sci. Transl. Med. **4**, 141ra92. Copyright 2012 American Association for the Advancement of Science AAAS.)

The classic Hall effect refers to the generation of a voltage difference (Hall voltage; *V*_H_) in an electrical conductor within a magnetic field. Moving charges, deflected by Lorenz force, accumulate on one side of the conductor to generate this voltage difference. Recently, Issadore et al. [18] introduced a new application of the Hall technology by developing a sensor to detect magnetically labelled CTCs in flow (Figure 3a). The sensor, known as MicroHall (μHall) platform, detects magnetic fields from MNP-labelled cells. The measured *V*_H_ was proportional to the MNP counts per cell to enable quantitative molecular analysis. Each sensor chip had μHall elements arranged into an overlapping 2×4 array, to ensure that each individual cells pass over at least two μHall elements (Figure 3b). In recent work, Muluneh and Issadore [55] demonstrated that multiple magnetic sensing chips could be integrated into a single microfluidic device, enabling incorporation of magnetic sensing with on-chip sample processing and parallel operation for improved throughput.

As compared with conventional flow cytometry, the μHall platform showed good agreement for molecular profiling (Figures 3c and 3d). Importantly, because the sensor is capable of detecting individual cells even in the presence of large abundance of blood cells, the μHall is well suited for rare cell detection in complex biological media. In a clinical trial with cancer patient blood samples, the μHall detected CTCs in all patient samples, even those that tested negative with the clinical standard (CellSearch™) [18].

## EXOSOMES FOR CANCER DETECTION

Exosomes have recently emerged as a new class of promising circulating biomarkers. Exosomes are membrane-bound phospholipid vesicles (30–150 nm in diameter) actively secreted by a variety of mammalian cells, especially dividing cancer cells [6,11] (Figure 1b). As compared with CTCs, exosomes exist in large quantities in biofluids, even in tumours (e.g., brain tumours) that release sparse numbers of CTCs. In addition, exosomes also carry diverse cellular constituents of their parent cells, including proteins [56], mRNA and miRNA [57,58], DNA [59], and have been shown to play various roles in modulating tumour microenvironment [60,61].

### Exosomes as disease markers

Exosomes offer significant advantages, in terms of abundance, stability and diversity, for cancer diagnostics and monitoring [6,62,63]. Exosomes have been identified in large quantities in a variety of bodily fluids, including blood, ascites and cerebrospinal fluid, even in tumours that do not release detectable quantities of CTCs [6,62,63]. For example, tumours of the central nervous system, lying behind a partially intact blood brain barrier, typically do not release many CTCs; however, a large quantity of cancer exosomes have been detected in blood samples of tumour patients [21,56,58]. As an abundant and stable source of circulating biomarkers, exosomes carry and protect diverse molecular information from degradation and can be analysed from banked and frozen biological samples [64].

With their rich contents of proteins and nucleic acids, exosomes can provide information on both the primary tumour and its microenvironment. Comparative protein analysis of circulating exosomes has identified a variety of disease-specific protein targets for solid tumours. These signatures include EpCAM for colorectal [65,66] and ovarian cancers [24,67], epidermal growth factor receptor (EGFR) for brain cancers [21,56] and human epidermal growth factor receptor 2 receptor (HER2/neu) for breast cancer [68].

In addition to proteins, exosomes also contain nucleic acids including different types of RNAs [57,58] and DNAs [59], all of which can be used as reflective disease markers. In a seminal work, Skog et al. [58] found that serum exosomes of glioblastoma patients contain characteristic mRNA mutant (EGFRvIII mRNA) and miRNAs that could be used to provide diagnostic information. Subsequent studies have since identified novel exosomal miRNA markers for a variety of tumours including lung cancer [69,70], prostate cancer [71,72] and gastric cancer [73], and demonstrated the utility of exosomal DNAs for tumour molecular analyses [59,74,75]

### Exosome challenges and detection technologies

The clinical translation of exosomes poses its unique sets of challenges, primarily because of the small dimension of exosomes. Conventional assays typically involve time-consuming ultracentrifugation to concentrate large volumes of samples and extensive processing for detection [11]. Current methods are therefore impractical for clinical applications, especially in studies that involve a large throughput or rare molecular targets.

To address these technological challenges, a series of miniaturized systems have been developed to facilitate exosome analyses [21–24,26,28,29]. New and improved strategies for exosome isolation, enrichment and identification will have marked implications in improving our understanding of exosome biology and in facilitating the translation of these biomarkers for better clinical outcomes.

#### Microfluidic enrichment

Microfluidic devices have been designed to collect intact exosomes directly from biological samples, replacing ultracentrifugation or proprietary precipitation methods. One such example is a device using a detachable membrane filter (0.1 μm pore) to size-selectively enrich exosomes from large sample volumes [22]. A capillary layer, inserted beneath the membrane, guides the filtered exosomes to the collection channel. The filter and guiding layer are sandwiched between two permanent ring magnets, which enable easy replacement of filter units when processing large sample volumes. More recently, an acoustic nanofilter system was demonstrated to separate exosomes in a continuous flow manner [28]. The separation is contact-free as it uses ultrasound standing waves to exert differential acoustic force on vesicles. Vesicles are thus separated according to their size and density, achieving high separation resolution and yield. With the capacity for rapid and efficient exosome isolation from small sample volumes, these developed microfluidic systems could become versatile preparatory platforms to accelerate the clinical translation of exosomes.

#### Magnetic detection

**Figure 4:**
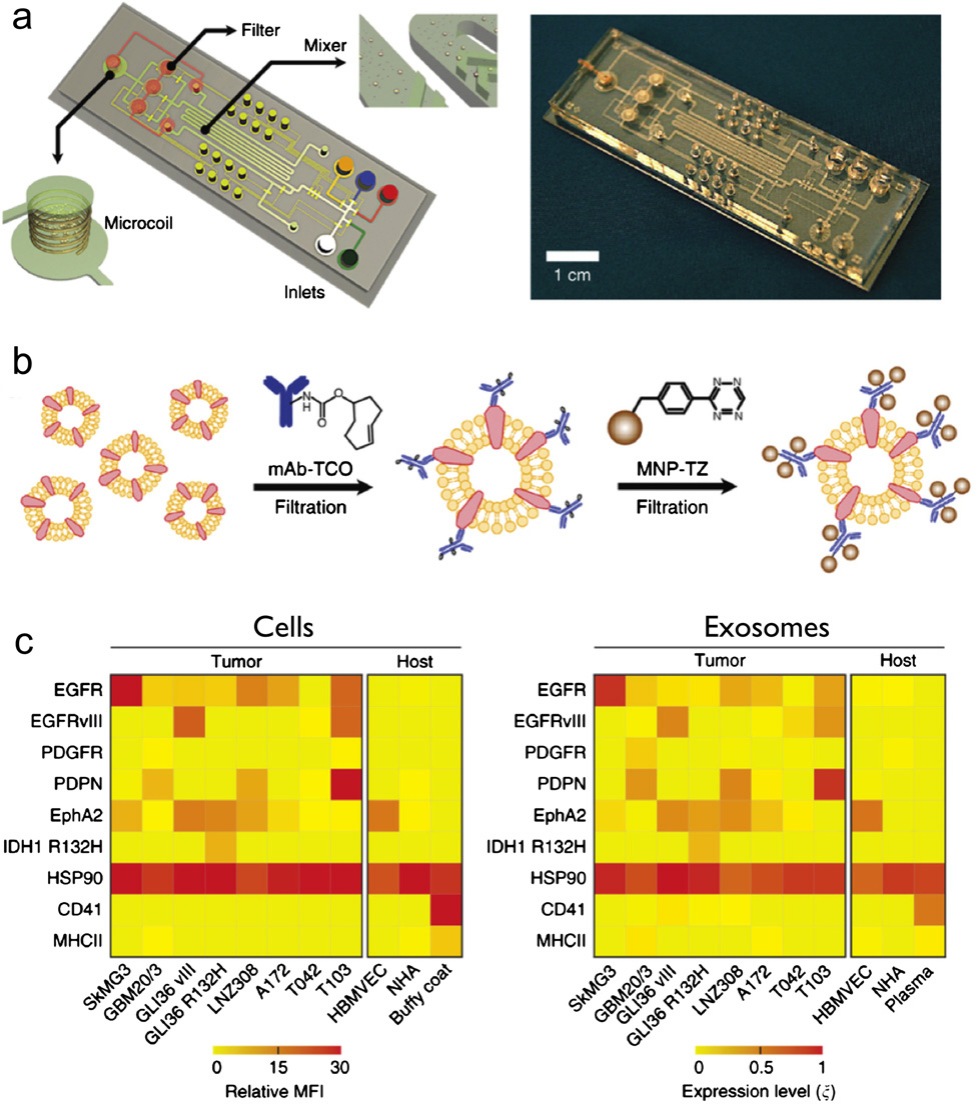
A microfluidic micro-nuclear magnetic resonance (μNMR) platform for magnetic detection of exosomes (**a**) The microfluidic system is designed to concentrate magnetically labelled exosomes and provide in-line NMR detection of exosomal protein markers. (**b**) Schematics of a two-step labelling procedure used to maximize MNP binding on to target proteins on vesicles (not to scale). (**c**) Comparative protein analyses confirmed that exosomes reflect the protein profiles of their parental cells and a distinct molecular signature can distinguish tumour exosomes from host cell-derived exosomes. MFI, mean fluorescence intensity; *ξ*, expression level defined by μNMR. (Adapted with permission from [21]: Shao, H., Chung, J., Balaj, L., Charest, A., Bigner, D.D., Carter, B.S., Hochberg, F.H., Breakefield, X.O., Weissleder, R. and Lee, H. (2012) Protein typing of circulating microvesicles allows real-time monitoring of glioblastoma therapy. Nat. Med. **18**, 1835–1840. Copyright 2012 Nature Publishing Group.)

As in CTC detection, magnetic technology can be an ideal tool for exosome protein analysis. The technology uses MNPs to label the vesicles through their protein markers; a microfluidic micro-nuclear magnetic resonance (μNMR) system is then used to determine the amount of biomarkers present by measuring the MNP concentration (Figure 4a).

The μNMR system had been used to detect whole tumour cells [14–16]. Adapting NMR to exosome detection, however, presented considerable engineering challenges because these vesicles are much smaller than tumour cells. A new analytical technology and a microfluidic system were thus devised specifically for exosome detection and profiling. In this assay, a two-step bio-orthogonal click chemistry was used for MNP labelling (Figure 4b). The magnetic labelling renders the vesicles superparamagnetic, which results in faster decay of the ^1^H NMR signal. The decay rate (*R*2) is proportional to the MNP concentration, thus enabling the quantification of target exosome protein concentration [21].

The technology was then applied for rapid profiling of exosomes derived from glioblastoma multiforme (GBM). Comparative analyses confirmed that exosomes indeed reflect the protein profiles of their parental cells and a distinct molecular signature was identified for GBM-derived exosomes (Figure 4c). In a pilot clinical test, the platform was applied to profile exosomes from blood samples of GBM patients and healthy controls, and demonstrated that exosomal protein profiling could enable accurate disease diagnosis and monitoring of treatment-induced changes [21].

## CONCLUSIONS

Circulating cancer biomarkers can provide new clinical opportunities for minimally invasive diagnostics and sequential monitoring. Due to their diverse nature, different types of circulating biomarkers pose unique challenges. For examples, CTCs are extremely rare and short-lived, thus requiring high-throughput enrichment against a complex biological background of host blood cells [48]. Exosomes, although an abundant and robust biomarker, require extensive processing due to their nanometre dimension [11]. Novel technologies uniquely designed to overcome these specific technical challenges are underway. By enabling rapid sample preparation and molecular analyses from small sample volumes, these platforms can help to fully harness the clinical potential of these circulating biomarkers in cancer and beyond.

## Abbreviations

CTC: circulating tumour cell
EGFR: epidermal growth factor receptor
EMT: epithelial-to-mesenchymal transition
EpCAM: epithelial cell adhesion molecule
μFCS: microfluidic cell sorter
GBM: glioblastoma multiforme
MNP: magnetic nanoparticle
μNMR: micro-nuclear magnetic resonance
